# Outbreak of Epidemic Keratoconjunctivitis Caused by Human Adenovirus Type 56, China, 2012

**DOI:** 10.1371/journal.pone.0110781

**Published:** 2014-10-24

**Authors:** Guohong Huang, Wenqing Yao, Wei Yu, Lingling Mao, Haibo Sun, Wei Yao, Jiang Tian, Ling Wang, Zhijian Bo, Zhen Zhu, Yan Zhang, Zhuo Zhao, Wenbo Xu

**Affiliations:** 1 National Institute for Viral Disease Control and Prevention, Chinese Center for Disease Control and Prevention, Beijing, People's Republic of China; 2 Liaoning Center for Disease Control and Prevention, Shenyang, Liaoning Province, People's Republic of China; 3 Dalian Center for Disease Control and Prevention, Dalian, Liaoning Province, People's Republic of China; 4 Clinical laboratory, People's Hospital of Xinjiang Uygur Autonomous Region, Urumqi, Xinjiang Uygur Autonomous Region, People's Republic of China; California Department of Public Health, United States of America

## Abstract

HAdV-56 is a new recombinant type isolated from epidemic keratoconjunctivitis (EKC) patients and has been sporadically isolated in Japan several times. Here, an outbreak of EKC in the city of Dalian, China involving a large number of workers in two factories was reported; this was the first outbreak of EKC associated with HAdV-56 worldwide.

## Introduction

Human adenoviruses (HAdVs) belong to the genus *Mastadenovirus* of the family Adenoviridae and are distributed worldwide [1]. A broad spectrum of clinical diseases are caused by HAdVs, including respiratory infectious diseases, conjunctivitis, gastroenteritis, cardiomyopathy and urinary tract infection [2]. More than 52 human adenovirus types are recognized and characterized within seven species (A- G). Of these, some types are commonly associated with conjunctivitis, such as HAdV-3 (in HAdV-B), -4 (in HAdV-E), -8, -19, -37,-53,-54 and -56 (all in HAdV-D). HAdV-8, -19 and -37 cause a more severe form of epidemic keratoconjunctivitis (EKC) than the others [3]–[5]. HAdV-53, -54 and -56 are the three new recombinant types that have been isolated from EKC patients and have been characterized by whole genome sequencing. EKC outbreaks caused by HAdV-53 and -54 have been reported in Japan several times, but HAdV-56 has only been sporadically isolated [6]–[9]. An outbreak of EKC in the city of Dalian in China involving a large number of workers in two factories become the first outbreak associated with HAdV-56 worldwide.

### The Study

**Figure 1:**
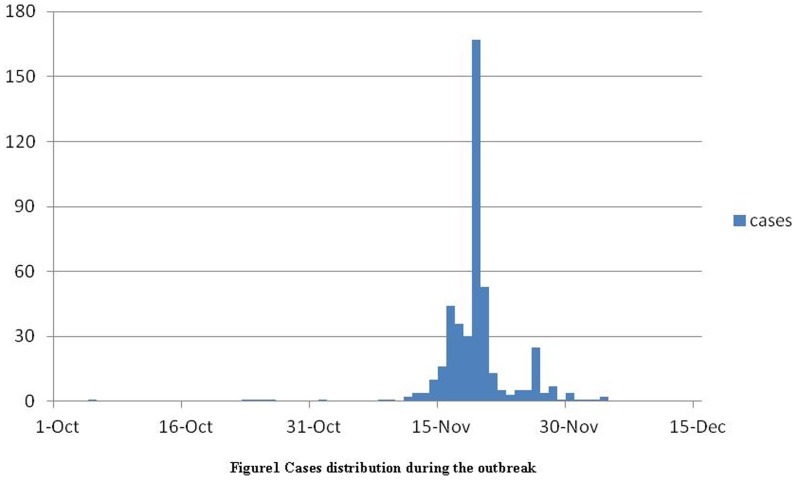
Cases distribution during the outbreak.

On October 5, 2012, a staff from Factory A in Dalian City, Liaoning Province, China, experienced pinkeye, itchiness, lacrimation in both eyes; additionally, herpes was observed. Three days later, he saw a doctor and antiviral treatment was given. Then he was on the sick leave from October 9 to October 22. His last contact with his colleagues was on the company shuttle bus without isolation. Subsequently, the number of employees from his factory presented with the similar symptoms increased gradually and pinkeye was their main symptom (Table 1). By December 5, 2012, a total of 451 patients from two factories (factory A and B) were infected. The incidence of the relevant cases can be seen in the bar graph (in Fig. 1).

**Table 1:** Main symptoms manifested in this outbreak (N = 451).

Clinical symptom	Number of cases	%
**Pinkeye**	411	91.13
**Secretion increase**	309	68.51
**Tearing (epiphora)**	260	57.65
**Soreness**	223	49.45
**Swelling**	221	49.00

The detailed information of Factory A and factory B is shown in Table 2. The factory buildings and their dormitory buildings are next to each other. In the dormitory buildings, a public toilet, wash room and hot water boiler are provided. The employees of these two factories share a bathroom on the basement level of building A, in which the lockers are public. The canteen includes the first and second floors of dormitory building B. The employees from different factories dine on different floors but share dishes, which are washed and disinfected together.

**Table 2:** Detailed information of the two factories.

	Factory A (%)	Factory B (%)
**Built-up area (m^2^)**	33231	106458
**Total number of employees**	2137	4807
**Male**	616(28.80)	1132(23.54)
**Female**	1521(71.2)	3675(76.45)
**Administrative staff**	501(23.4)	1007(20.94)
**Worker**	1636(76.6)	3800(79.05)
**Number of employees living in dormitories**	1355(63.4)	2425(50.44)
**Male**	445(20.8)	452(9.40)
**Female**	910(42.6)	1973(41.04)
**The average number of employees in a dormitory**	4–6	4–8

	Factory A (attack rate,%)	Factory B (attack rate, %)
Total number of patients*	250(11.70)	201(4.18)
Male*	92(14.94)	25(2.21)
Female*	158(10.39)	176(4.79)
Administrative staff*	10(2.00)	9(0.89)
Worker*	240(14.67)	192(5.05)
Number of patients living in dormitories*	232(17.12)	188(7.79)

“*” The attack rate between Factory A and B was significantly different.

All the patients recovered quickly after treating for their symptoms, and the prognosis was good. No severe cases associated with this outbreak occurred. Isolation and infection control measures were taken to prevent the spread of disease.

## Materials and Methods

### Specimen Collection

This study did not involve human participants or human experimentation; the only human materials used were conjunctival swabs collected for infectious disease outbreak control and interpretation purposes from the patients with epidemic keratoconjunctivitis. Written informed consent for the use of the clinical samples was obtained from all patients involved in this study. This study was approved by the second session of the Ethics Review Committee of the National Institute for Viral Disease Control and Prevention, Chinese Center for Disease Control and Prevention.Staffs from the Liaoning Center for Disease Control and Prevention (Liaoning CDC) collected 36 conjunctival swab specimens from 36 patients (18 patients from Factory A and 18 patients from Factory B). The specimens were collected in 2-mL viral transport media, transported at 2°C–8°C, and preserved at −80°C. The first round of studies using real-time PCR was conducted at the Liaoning CDC, and the second round was conducted at the Institute for Viral Disease Control and Prevention at the Chinese Center for Disease Control and Prevention, Beijing.

### Virus Isolation, PCR, and Sequencing

HAdV was isolated by using the Hep-2 cell line, and the infected cells were harvested when >75% of the culture showed a cytopathic effect (CPE). Meanwhile, DNA was extracted from all clinical specimens using the QIAamp DNA mini kit (Qiagen, Valencia, CA, USA) according to the manufacturer's instructions. PCR was performed to amplify the hypervariable region (HVR1-6) of the hexon gene by using the primers to detect all HAdV types [10], the partial fiber gene and the genome. The primers used in this study were designed by Primer 3 (v. 0.4.0) and are shown in Table 3. The PCR products were purified using a QIAquick Gel Extraction kit (QIAGEN). Sequences of the amplicons were obtained by using BigDye terminator version 3.0 chemistry according to the manufacturer's protocol. The ABI PRISM 3100 DNA Sequencer (PerkinElmer, Beijing, China) was used. The sequences were analyzed using Sequencer (Gene Codes Corporation, Ann Arbor, MI, USA) and version 7.0 of BioEdit (www.mbio.ncsu.edu/BioEdit/BioEdit.html). The phylogenetic analyses were performed, and trees were generated using MEGA4 (www.megasoftware.net). The robustness of the groupings was assessed using bootstrap resampling of 1,000 replicates.

**Table 3:** The primers for HAdV-D genome*.

Name	Sequence(5′-3′)
1s	GTTTCGATTGCGGTGTTTTT
1a	GGTGTTCTGGCTCCATTTGT
2s	CTCCTCTATCTCGCCTGGTG
2a	CACCCTGGACTTGGTCTCAT
3s	GGGTCTGCCATATCCTGAAG
3a	TCCCAGCTGTTGAGAAACCT
4s	ATGCTCCTGGGTGAGATCAT
4a	CAGGCCAACATCAAATCCTC
5s	GGTCTGTGCTCGTCCTCACT
5a	ACGAGGAGCTCAAAAAGCTG
6s	GGTTAGGCAGAGCGAAAGTG
6a	GTCGCATCTCGAATGACCTC
7s	CCCGTGACTTTGAACCTGAA
7a	ATGGACGACTTCCAGGACAC
8s	ATGAGCCTCTCGATGTCATCACTGG
8a	GCCCTCTTCCAAGTCCATCT
9s	CCAGCCGACTTCTCCAGTTA
9a	CCTCCTCCTCTTCCTCATCC
10s	TCAAGGTGCTGACGCTGA
10a	TCAGACTGTCGGTGTCGTTC
11s	GTCTGGCCCGGACTACTTTT
11a	GCTATGGCCTTGGTGAGTTT
12s	TGGCCAATAACGGGATAGAGAGTC
12a	GATTCTGAAGCCCTCTTGGAAAGG
13s	CGGTGACCAAGCTGGTGATGCC
13a	CGCTTCTTGGCTCCTCCGTACAT
14s	CTCCCTCACCCACGTCTTC
14a	AAACTCTACTGCCCCCTCGT
15s	TCCTCCTCGTCCCTGATCTA
15a	TAAAGGATGCTGGGGTGGTA
16s	GTTGCATCCTTCCATCATCC
16a	GATACTGTGCAGCGTGCTCT
17s	GGTGGTGGAGATGGAAGATG
17a	GGGTCTTGTTTGCCATTTGT
18s	GTACAAGGCGCGCTTCACT
18a	GTAGGAGTCGGGCAGGTACA
19s	CATTGAGAATCACGGTGTGG
19a	GGAGTACATGCGGTCCTTGT
20s	TTGTCTACTCGGGCTCCATC
20a	CAAGTTCATGGTGGGGTTTC
21s	GCACCTGCTACATGTTCGAC
21a	GCTCAAGTGTCTGCATGGAA
22s	CGCTGCATATTCGGGTAGAG
22a	TGTTTCAGCAGCACATCCTC
23s	CCTAGGGGAAGATGGAGGAG
23a	TTGTCCAGCTCCTTCAGGTT
24s	TTCAGACACGGTTTCGTCAG
24a	TCGATCCCATCTACGAGGAG
25s	ATGGAAGAAGAATGGGACAGC
25a	TCTGCAAGGTCAGGTAGGCCTGG
26s	GCCCACAATCGGGTATAAAA
26a	CCCACCAGGGAAAGGATAAT
27s	CGTTTCGGAGGTGAGCTAAG
27a	GCAGTGTCAGGTTCTGGATG
28s	AAGCCTTTGTCAGAGCCTCA
28a	TGCAGTAACATTCCATTCACAG
29s	GATTGCGGGCTTTGTAACTC
29a	CCTGTATTCGGGACTGTGGT
(28–29)s	GATGGCGAACTTAATGATCCA
(28–29)a	AATGTTGGCAGTGGCAGTAA
30s	CAGTAATCGGGGAACCTCAG
30a	TGTGGACCAGGAATTTGACA
31s	CTCCCTCCTCAAGACCCTTT
31a	GTGGTAGGTTTTGGATACGC
32s	AGGTGGCGGCTTATCATTTA
32a	CATCTGGTACGATGGTCACG
33s	CACAGCCTTCAGCATCAGTC
33a	CCAAGGCTGGAAAAATGAGA
34s	CTCTACGCGTACATCCAGCA
34a	AAGTGAGAACGCGGAAGTGT
35s	GTGGAAAGATTCAGGGCAAA
35a	AAGCGGTGATGCAAATGAC
36s	TGCAAATGAGCTTTTGAATTTT
36a	TCTCTACTCGCTGGCACTCA

* All the primers were designed to amplify whole genome (except the 3′ and 5′ termination (GenBank accession number: HM770721).

### Statistical analysis

The descriptive statistics and Pearson's test were used in this study (α = 0.01).

## Results

Since the index patient appeared on October 5, 451 patients were infected with similar symptoms by December 5 (Table 1). The patients ranged in age from 18 to 43 years, with a median age of 24. Based on the incidence of the relevant cases (Fig. 1), a person-to-person transmission pattern occurred in this outbreak, and the presumed incubation period was approximately 10–15 days.

Out of the 451 patients, 250 patients were from the same factory as the index patient, and 201 patients were from the neighboring factory (Factory B). The attack rate of Factory A (11.70%, 250/2137) was higher than Factory B (4.18%, 201/4807) (P<0.01). The high attack rate in workers (7.95%, 432/5436) was found to be significantly higher than that of administrative staffs(1.26%, 19/1508) (P<0.01). A total of 117 male patients (attack rate of 6.69%, 117/1748) and 334 female patients (attack rate of 6.43%, 334/5196) were infected, but no significant difference was found. The details of the patients in these two factories are shown in Table 2.

At the beginning of the outbreak described here, the disease was suspected to be herpes simplex keratitis. Real-time PCR was performed with 21 clinical specimens to detect the herpes virus and HAdVs. Positive results were obtained for only HAdV. PCR was performed with a total of 36 clinical specimens and specific primers for HVR1-6 of the HAdV hexon gene; 35 of the 36 specimens were positive. A BLAST sequence analysis revealed that the 35 positive HAdV were 100% identical with HAdV-56 (GenBank accession number: HM770721).

All 36 clinical specimens were separately inoculated into Hep-2 cells, and a characteristic adenovirus-like CPE was observed in the Hep-2 cells for 5 conjunctival swab samples. In all of these cases, a CPE was observed within three passages after inoculation.

**Figure 2:**
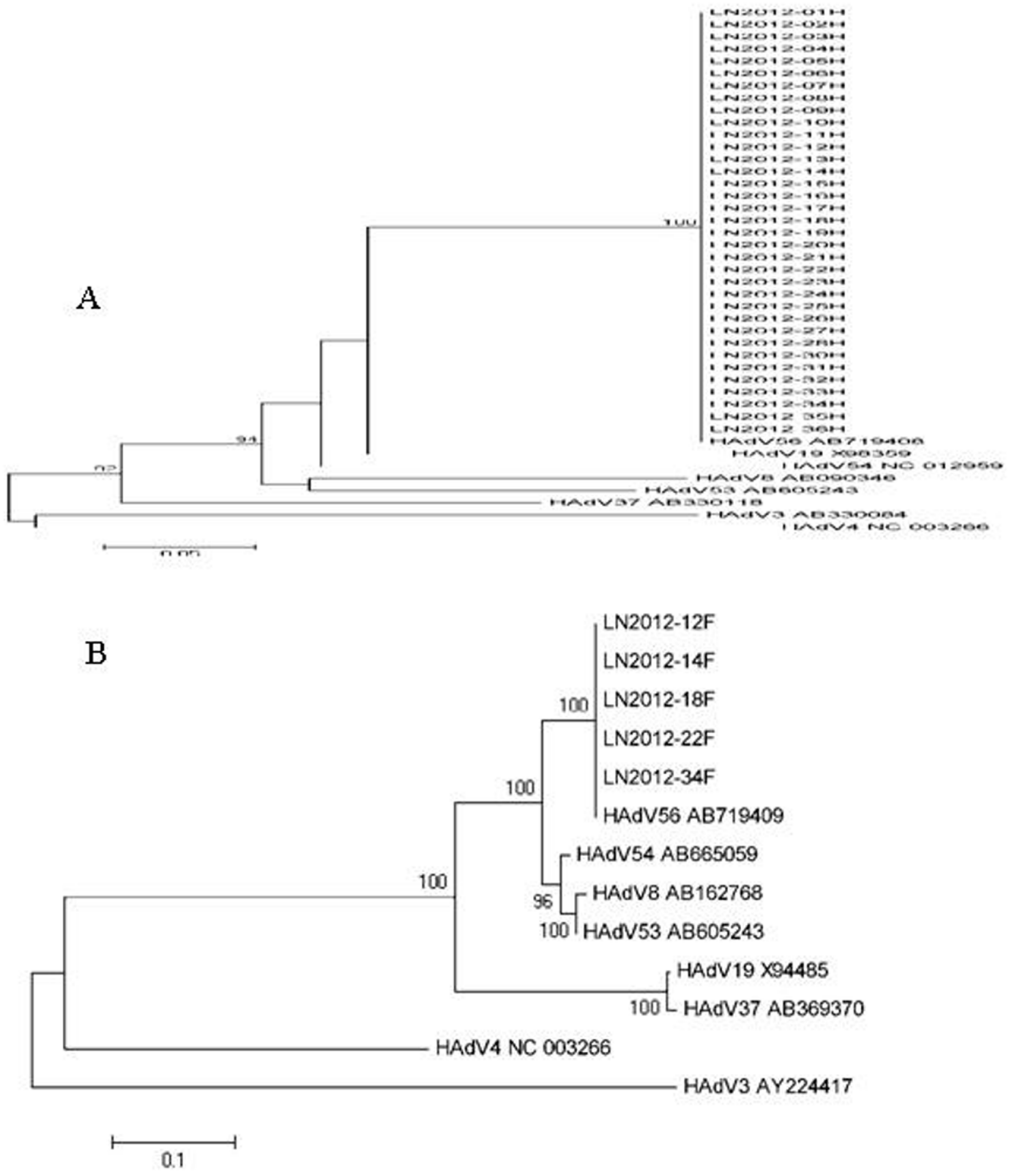
Phylogenetic analysis of the partial hexon and fiber genes of HAdV-56 in this outbreak by the neighbor-joining method. (A) Phylogenetic tree based on the partial hexon gene of the HAdV-56 Liaoning 2012-01∼36H strains and reference strains of other common HAdVs that cause EKC. (B) Phylogenetic tree based on the partial fiber gene of the HAdV-56 strains (Liaoning 2012-12, 14, 18, 22 and 34F) and reference strains of other common HAdVs that cause EKC.

DNA from 5 HAdV isolations was successfully amplified by PCR with primers specific for the partial fiber gene of HAdV-D. The phylogenetic trees constructed on the basis of the partial hexon gene (789 nt) and the fiber gene (858 nt) nucleotides (Figure 2), which revealed that all PCR-positive samples belonged to the species HAdV-D and were in the same cluster as HAdV-56. Therefore, we concluded that all the patients were infected with the same virus. 35,036 bp of the whole genome was amplified (except for the 3′ and 5′ termination) (GenBank accession number: KF302429); the results showed 99.3% identity with HAdV-56 (GenBank accession number: HM770721).

## Discussion

HAdVs associated with EKC are common worldwide, especially HAdV-8, 19, and 37 [11]–[14]. Our findings provide the first data of HAdV-56 as an agent in an EKC outbreak worldwide.

HAdV-56 was first identified in a 10-day-old neonate who died from fatal pneumonia. Ten days after death, three health care providers developed EKC [15]. Subsequently, sporadic isolations of HAdV-56 from patients with pharyngoconjunctival fever and urethritis had occurred in Japan [8]. Additionally, surveillance of HAdV- D in patients with epidemic keratoconjunctivitis was performed in Japan, and HAdV-56 was detected as one of the pathogens [9]. Since 2009, HAdV-56 was identified as a new agent several times, but this paper describes the first outbreak of HAdV-56 in China.

EKC is a highly contagious infectious disease that mainly involves the surface of the eye. All age groups can be infected, and the disease can occur in the whole year. HAdVs as a pathogenic agent are highly resistant to environmental influences and are transmitted from person to person by secretions. Transmission often occurs in crowded places, such as schools, factories and people with low immunity [16]. The outbreak in this study chanced to be in factories with large populations and most of the patients were the workers living in the dormitories, in which both their working and living environments were closed and crowded. On the other hand, bad hygienic habits and environments increased transmission quickly. Though the local government attached great importance to this emergent public health event, it still caused stoppages in the partial departments and sizable economic losses to the factories.

From this outbreak, we could conclude that: (1) through the retrospective investigation, the index case was found and described. However, optimal opportunity of sample collection had been lost. Due to the lack of the laboratory test evidence and the typical clinical symptoms of EKC, this index case was identified as a suspected EKC cases. Soon after, the following patients from his factory presented the similar symptoms to the index patient and identified as HAdV-56 infection with lab confirmed. Therefore, this index case was not incidental and directly associated with the larger outbreak with epidemiological data support. (2) the clinical characteristics caused by HAdV-56 were similar to the other HAdVs, no more additional serious symptoms were observed. The recombination in the genome of HAdV-56 may not change its virulence; (3) the incubation period of EKC caused by HAdV-56 was presumed to be approximately 10-15 days; this may provide a basic reference for prevention and control measures; (4) according to the data in Table 2, most of workers in Facory A lived in dormitory may lead to the higher attack rate in Factory A. As mentioned, crowded environment can contribute to the spread of virus.

HAdV-56 has been identified in France, Japan [6]–[9], [17] and Thailand [18]; and it was also detected in China during this EKC outbreak in 2012. As the number of samples collection in this study was limited, it is unclear that all 451 patients were infected with HAdV-56. However, as all the patients presented the similar symptoms and had the epidemiological link, HAdV-56 was recognized as the pathogen responsible for this outbreak. In light of the ability for multiple-recombination in HAdV-56 and the lack of knowledge of its virulence and pathogenicity, much attention should be paid to this virus.
